# Supplementary material for: Resolving the full spectrum of human genome variation using Linked-Reads
Source: Genome Res. 2019 Apr;29(4):635–45. doi: 10.1101/gr.234443.118 (PMC6442396; doi:10.1101/gr.234443.118)
Supplement: Supplemental Material [file supp_gr.234443.118_Supplemental_longranger-2.2.zip › longranger-2.2.tar.bz2!/lr2.2-public/lib/stan/cmdstan/stan/doc/api/html/check__symmetric_8hpp_source.html]

Stan: src/stan/math/error\_handling/matrix/check\_symmetric.hpp Source File


|  |  |
| --- | --- |
|  | Stan  2.5.0  probability, sampling & optimization |


- Main Page
- Related Pages
- Namespaces
- Classes
- Files

- File List
- File Members

All Classes Namespaces Files Functions Variables Typedefs Enumerations Enumerator Friends Macros Pages

- src
- stan
- math
- error\_handling
- matrix

check\_symmetric.hpp

Go to the documentation of this file.

1 #ifndef STAN\_\_MATH\_\_ERROR\_HANDLING\_\_MATRIX\_\_CHECK\_SYMMETRIC\_HPP

2 #define STAN\_\_MATH\_\_ERROR\_HANDLING\_\_MATRIX\_\_CHECK\_SYMMETRIC\_HPP

3

4 #include <sstream>

5

6 #include <stan/math/error\_handling/dom\_err.hpp>

7 #include <stan/math/error\_handling/matrix/constraint\_tolerance.hpp>

8 #include <stan/math/matrix/Eigen.hpp>

9 #include <stan/math/matrix/meta/index\_type.hpp>

10 #include <stan/meta/traits.hpp>

11

12 namespace stan {

13

14  namespace math {

15

29  template <typename T\_y, typename T\_result>

30  inline bool

31  check\_symmetric(const char\* function,

32  const Eigen::Matrix<T\_y,Eigen::Dynamic,Eigen::Dynamic>& y,

33  const char\* name,

34  T\_result\* result) {

35  using Eigen::Dynamic;

36  using Eigen::Matrix;

37  typedef typename index\_type<Matrix<T\_y,Dynamic,Dynamic> >::type size\_type;

38

39  size\_type k = y.rows();

40  if (k == 1)

41  return true;

42  for (size\_type m = 0; m < k; ++m) {

43  for (size\_type n = m + 1; n < k; ++n) {

44  if (!(fabs(y(m,n) - y(n,m)) <= CONSTRAINT\_TOLERANCE)) {

45  std::ostringstream message;

46  message << name << " is not symmetric. "

47  << name << "[" << stan::error\_index::value + m << ","

48  << stan::error\_index::value +n << "] is %1%, but "

49  << name << "[" << stan::error\_index::value +n << ","

50  << stan::error\_index::value + m

51  << "] element is " << y(n,m);

52  std::string msg(message.str());

53  return dom\_err(function,y(m,n),name,

54  msg.c\_str(),"",

55  result);

56  }

57  }

58  }

59  return true;

60  }

61

62  }

63 }

64 #endif

dom\_err.hpp

stan::agrad::fabs

fvar< T > fabs(const fvar< T > &x)

**Definition:** fabs.hpp:18

stan::math::dom\_err

bool dom\_err(const char \*function, const T &y, const char \*name, const char \*error\_msg, const T\_msg error\_msg2, T\_result \*result)

**Definition:** dom\_err.hpp:36

constraint\_tolerance.hpp

traits.hpp

stan::math::CONSTRAINT\_TOLERANCE

const double CONSTRAINT\_TOLERANCE

The tolerance for checking arithmetic bounds In rank and in simplexes.

**Definition:** constraint\_tolerance.hpp:11

stan::math::check\_symmetric

bool check\_symmetric(const char \*function, const Eigen::Matrix< T\_y, Eigen::Dynamic, Eigen::Dynamic > &y, const char \*name, T\_result \*result)

Return true if the specified matrix is symmetric.

**Definition:** check\_symmetric.hpp:31

stan::math::index\_type

Primary template class for the metaprogram to compute the index type of a container.

**Definition:** index\_type.hpp:21

stan::error\_index::value

**Definition:** traits.hpp:20

index\_type.hpp

Eigen.hpp

stan::math::size\_type

index\_type< Eigen::Matrix< double, Eigen::Dynamic, Eigen::Dynamic > >::type size\_type

Type for sizes and indexes in an Eigen matrix with double e.

**Definition:** typedefs.hpp:16


---

[ Stan Home Page ]

*© 2011–2014,
Stan Development Team.*
